# Identification and quantitation of 2´,3´-cGMP in murine tissues

**DOI:** 10.1186/2050-6511-14-S1-P12

**Published:** 2013-08-29

**Authors:** Heike Burhenne, Sarah Tschirner, Roland Seifert, Volkhard Kaever

**Affiliations:** 1 Institute of Pharmacology, Hannover Medical School, Germany; 2 Research Core Unit Mass Spectrometry - Metabolomics, Hannover Medical School, Germany

## Background

**Figure 1:**
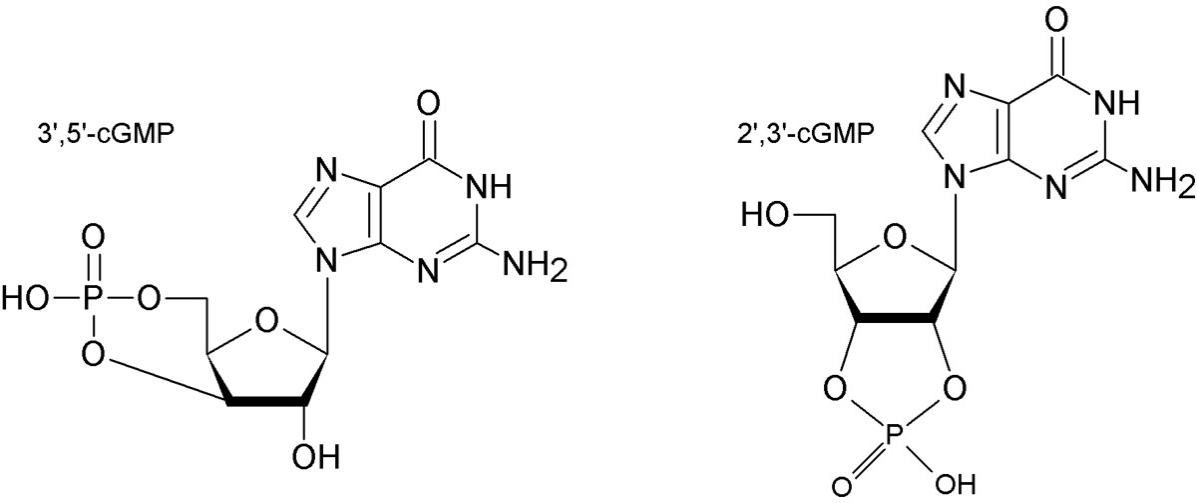
Chemical structures of 3’,5’-cGMP and 2’,3’-cGMP

3´,5´-cGMP is a well-known second messenger in eukaryotic cells which is synthesized by particulate and soluble guanylyl cyclases and is involved in several cardiovascular and neurological processes [1,2]. It has been recently reported that also **2´,3´**-cGMP (Figure 1) can be found in high concentrations in rabbit kidney and pancreas [3]. So far, little is known about the source and biological role of 2´,3´-cGMP as well as its tissue distribution. To address this question, we have developed sensitive and specific liquid chromatography-coupled mass spectrometry (LC-MS/MS) methods for identification and quantitation of 2´,3´- nucleoside monophosphates (2´,3´-cNMPs) simultaneously with 3´,5´-cNMPs (cAMP, cCMP, cGMP, cUMP). We systematically analyzed mouse tissues (brain, thymus, heart, lung, liver, pancreas, spleen, kidney, bladder, testis, ovary, uterus) for the presence of 2’,3’- and 3’,5’-cNMPs, respectively.

## Materials and methods

For cNMP extraction, 30-200 mg tissue was transferred to a 2.0 mL tube containing a garnet matrix and one ¼ inch ceramic sphere. An organic extraction solvent was added and tissues were homogenated in a FastPrep-24^®^ system (MP Biomedicals, Germany). The homogenate was centrifuged and the supernatant fluid was evaporated to dryness at 40°C under a nitrogen stream. The residue pellet was resuspended in water and analyzed by LC-MS/MS.

Detection and quantitation of 2’,3’- and 3’,5’-cNMPs was performed on a tandem mass spectrometer (5500 QTRAP^®^; AB SCIEX, USA). We confirmed our data on a TripleTOF^TM^ 5600 system (AB SCIEX, USA) which is characterized by an extremely high mass accuracy.

## Results

**Figure 2:**
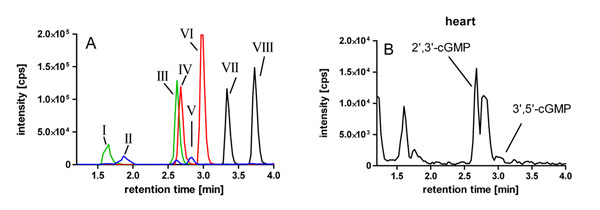
**A**: Representative chromatogram of a 3’,5’- /2’,3’ cNMP standard (I: 2’,3’-cCMP, II: 2’,3’-cUMP, III: 3’,5’-cCMP, IV: 2’,3’-cGMP, V: 3’,5’-cUMP, VI: 3’,5’-cGMP, VII: 2’,3’-cAMP, VIII: 3’5’-cAMP) **B**: Detection of 2’,3’-cGMP and 3’,5’-cGMP in murine heart

The chromatogram of a standard cNMP sample demonstrates that the LC-MS/MS method is suitable for the detection and quantitation of 2’,3’- and 3’,5’-cNMPs. Due to their retention times, all 2’,3’-cNMPs could be reliably discriminated from their 3’,5’-isomers (Figure 2A).

In addition to 3’,5’-cGMP we detected notable amounts of 2’,3’-cGMP in various mouse tissues. For example, heart samples showed an up to 5-fold higher concentration of 2’,3’-cGMP compared with the 3’,5’-cNMP (Figure 2B). In murine pancreas and spleen only 2’,3’-cGMP but no 3’,5’-cGMP could be detected.

Besides 2’,3’-cGMP, remarkably high levels of 2’,3’-cCMP and 2’,3’-cUMP were detected in murine heart, kidney, spleen, liver, pancreas and lung.

## Conclusion

2’,3’-cAMP is an mRNA degradation product. Increased 2’,3’-cAMP concentrations may play a role in cell death and cell proliferation [4].

We have demonstrated that high levels of 2’,3’-cGMP as well as 2’,3’-cCMP and 2’,3’-cUMP can be detected in various murine tissues. Our results indicate that these cNMPs play an important but still unknown role in (patho)physiological processes.

To our knowledge, this is the first time that mammalian tissues were systematically analyzed for the occurrence of 2’,3’-cNMPs. Our methods allow reliable detection and quantitation of four 2’,3’-cNMPs simultaneously with their 3’,5’-isomers and are, therefore, useful for the characterization of the physiological role of 2’,3’-cNMPs.

## References

[B1] Kots AY Martin E Sharina IG Murad F A short history of cGMP, guanylyl cyclases, and cGMP-dependent protein kinases Handb Exp Pharmacol 2009 191 1 14 10.1007/978-3-540-68964-5_1 19089322 PMC3932363

[B2] Stasch JP Pacher P Evgenov OV Soluble guanylate cyclases as an emerging therapeutic target in cardiopulmonary disease Circulation 2011 123 2263 2273 10.1161/CIRCULATIONAHA.110.981738 21606405 PMC3103045

[B3] Van Damme T Zhang Y Lynen F Sandra P Determination of cyclic guanosine- and cyclic adenosine monophosphate (cGMP and cAMP) in human plasma and animal tissues by solid phase extraction on silica and liquid chromatography-triple quadrupole mass spectrometry J Chromatogr B 2012 909 14 21 23153638 10.1016/j.jchromb.2012.10.002

[B4] Jackson EK The 2´,3´-cAMP-adenosine pathway Am J Physiol Renal Physiol 2011 301 F1160 F1167 10.1152/ajprenal.00450.2011 21937608 PMC3233866

